# Fabrication of three dimensional patterns of wide dimensional range using microbes and their applications

**DOI:** 10.1038/srep15416

**Published:** 2015-10-21

**Authors:** Sunita Mehta, Saravanan Murugeson, Balaji Prakash, Deepak

**Affiliations:** 1 Department of Materials Science & Engineering and Samtel Center for Display Technologies, Indian Institute of Technology Kanpur, Kanpur-208016, India; 2 Department of Biological Sciences & Bioengineering, Indian Institute of Technology Kanpur, Kanpur-208016, India

## Abstract

Inspired by the wound healing property of certain trees, we report a novel microbes based additive process for producing three dimensional patterns, which has a potential of engineering applications in a variety of fields. Imposing a two dimensional pattern of microbes on a gel media and allowing them to grow in the third dimension is known from its use in biological studies. Instead, we have introduced an intermediate porous substrate between the gel media and the microbial growth, which enables three dimensional patterns in specific forms that can be lifted off and used in engineering applications. In order to demonstrate the applicability of this idea in a diverse set of areas, two applications are selected. In one, using this method of microbial growth, we have fabricated microlenses for enhanced light extraction in organic light emitting diodes, where densely packed microlenses of the diameters of hundreds of microns lead to luminance increase by a factor of 1.24X. In another entirely different type of application, braille text patterns are prepared on a normal office paper where the grown microbial colonies serve as braille tactile dots. Braille dot patterns thus prepared meet the standard specifications (size and spacing) for braille books.

Many engineering applications are enabled by developing three dimensional (3D) patterns. To obtain such patterns, at first a two dimensional (2D) pattern is generated and another process is employed for producing the third dimension. Several micro-fabrication techniques are available for this purpose. Lithography is the most commonly used process to produce 3D patterns. In this, a photo or radiation-sensitive material is used to create the initial 2D pattern (having a small height, 100 nm to few μm), either by exposure of radiation through a mask or by a focused beam guided by a computer aided design^1^,^2^,^3^. Such patterns can also be obtained through nonconventional methods such as soft lithography or nano-imprinting^4^,^5^,^6^. Following this, a wet or dry etching process is used to achieve the third dimension. Such subtractive process involves expensive machinery, use of harsh chemicals and limited to selected substrates.

Similarly, methods such as laser machining and focused ion beam lithography (FIB) are capable of directly removing materials in a pattern^7^,^8^, with the latter also having capacity to directly depositing a few materials whose ion beam can be generated. Printing methods such as screen printing, flexography, gravure or ink-jet printing have also been employed for generating 2D patterns^9^,^10^,^11^. Among these methods, maximum height of the patterns is by screen printing, but still limited to 10–20 μm. Hence, to produce deeper 3D patterns, the patterns generated by these methods also are made to serve as protective layer and after that subsequent etching is still as in photolithography.

With the exception of FIB method and possibly 3D printing^12^ all other methods require a subtractive route to generate the third dimension for 3D structured patterns. However, these methodologies are not commonly used when the requirement for the third dimension exceeds 100 μm. Here, we are proposing a practical additive technique for producing 3D patterns, which is relatively easy in processing and likely to be significantly inexpensive for 3D patterns of significant height. Apart from that, there is simplicity in the method.

We were inspired by the special healing attribute in a tree named *Pongamia Pinnata* (also known as *Millettia pinnata*) that when a scratch is made on its trunk, it recovers over time as a protrusion rather than as an indentation (see Fig. 1). Therefore, we examine if a similar phenomenon can be realized by an equivalent natural process and put to use in engineering applications.

The central questions are: (a) can we mimic this uncommon natural phenomenon, shown in Fig. 1, in a laboratory, (b) can the pattern be grown in a practical time scale and dimensions of a few hundred micrometers or less?

To address these questions, one approach may be to examine the mechanisms specific to this tree, reproduce it within a laboratory setting and then find ways to expedite the rates and control the sizes. We have found an alternative. Microbes that are routinely used in a biological laboratory for basic research can grow in relatively short time scales. Therefore, we use them after engineering their 2-D patterns - a means different from protrusions seen upon scratching the bark of a tree.

The property to grow in three dimensions is prevalent in most living cells. Patterns of microbes such as *Escherichia coli*, *Staphylococcus aureus*, *Pseudomonas aeruginosa* have been obtained by laser assisted direct writing, lithography, inkjet printing and contact printing among other methods^13^. Patterns created thus were used to study phenotypic behavior, intercellular interactions and to further our understanding of various biological phenomenon at cellular and tissue level. However, it was not possible to use such patterns in engineering applications.

We would demonstrate different processes to control the growth of micro-organisms and a modified methodology which would enable useful 3D patterns, in height as much as 100 μm. The range and scope of these patterns will then be demonstrated in two applications– micro-lenses and Braille, which are briefly described first.

The micro-lenses of varying sizes and shapes have been used for increasing the extraction efficiency of organic light emitting diodes (OLEDs). The most commonly used method to fabricate them is lithography, which involves preparing a master in negative lens pattern on silicon and subsequently obtaining the microlens pattern on PDMS using replica molding^14^. In our work, we demonstrate that microbial patterns can be used to prepare masters for microlenses. A densely packed array of micro-lenses have been achieved using microbial master prepared from one of the processes used.

As opposed to micro-lenses, braille patterns are in millimeter scale. To achieve patterns of such dimensions, conventional braille printers use a thick paper on which protrusions are made using embossing pins in the form of braille patterns^15^. We show a process by which ordinary office paper can be used to create tactile microbe based braille dots. These two examples are selected to demonstrate a wide applicability of the process- in one, as an alternative to where photolithography is used and in the other, where mechanical pressure is used.

## Process development

Given optimum environmental conditions and abundant nutrients, most micro-organisms are capable of showing growth within a few hours. When inoculated on an agarified nutrient media (that acts as the substrate for growth), they grow to form physically distinct structures called colonies. Different microbes form colonies of different height and three-dimensional shape. Such microbial colonies have been patterned and reported in the literature^13^. However, the agar substrate used to develop these patterns is fragile in nature and imposes limitation on application of the patterns. Thus, the patterns of microbes have only been used for biological studies and no engineering applications have been shown so far.

The key to countering these challenges by growing the microbes on a thin porous substrate instead of agar. They permit uptake of nutrients through it for the growth of microbes when placed over agarified nutrient media. Such substrates are believed to act similar to agar for supporting microbial colony formation. The following criteria are deemed important for selecting the substrate: 1) it should exhibit optimal porosity suitable for both diffusion of nutrients and microbial colony formation; 2) it should delaminate easily from the agarified media without disturbing the microbial patterns present on it; 3) after delamination, it should sustain the microbial patterns when subjected to different processing steps required by the application. Membranes typically used in biochemistry laboratories such as RC membrane and PVDF membrane were found to be good candidates for this process.

**Figure 2:**
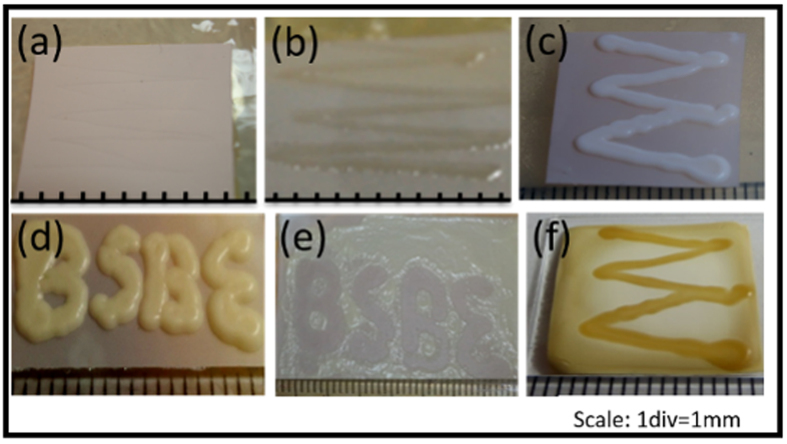
Photographic images for patterned growth of microbes on substrates. (**a**) untreated PVDF membrane displaying no growth (**b**) growth of E.coli on PVDF membrane treated with Triton X-100 (**c**) as-grown yeast on surfactant treated PVDF (**d**) legible pattern of grown yeast on PVDF membrane with culture ink (inoculum + surfactant) dispensed using micro-pipette (**e**) negative image of the same pattern in which culture ink is spread thorough-out and growth is inhibited by antimicrobial agent dispensed in the BSBE pattern. (**f**) grown yeast pattern with significant height even after drying.

The ability of these membranes to support the growth of *Saccharomyces cerevisiae* (Baker’s yeast), *Escherichia coli* (strain DH5-α) and *Enterococcus faecalis* (ATCC 19433) was tested. They were inoculated on the membrane substrates which were placed over agarified nutrient media and their growth was observed (see Methods). All three microbes displayed growth over RC membranes in defined patterns similar to microbes growing on agar. In contrast, no growth was observed on PVDF membrane (see Fig. 2a). Untreated PVDF membrane is highly hydrophobic, which may cause it to act as a poor substrate for adherence of microbes for growth (see Fig. 2a and Supplementary Fig. S1a online). Additionally, growth may be hindered because of poor diffusion/percolation of hydrophilic nutrients through it thereby limiting their availability to the microbes on the other side. This limitation can be circumvented by functionalizing the PVDF membrane using surfactants. Surfactants are generally known to hinder microbial growth, so Triton X-100 (concentrations as low as 0.1% v/v) - a mild non-denaturing surfactant (known to be a soft detergent) not severely detrimental to microbial cell viability was used^16^,^17^. It supported growth of both strains DH5-α (see Fig. 2b) and ATCC 19433, as well as the baker’s yeast (see Fig. 2c).

## Microbial patterning on membrane substrates

Patterning of microbes can be done by providing growth supporting agents or growth inhibitors on the membrane substrates in defined patterns. These two approaches are termed as microbial approach and anti-microbial approach.

**Figure 3:**
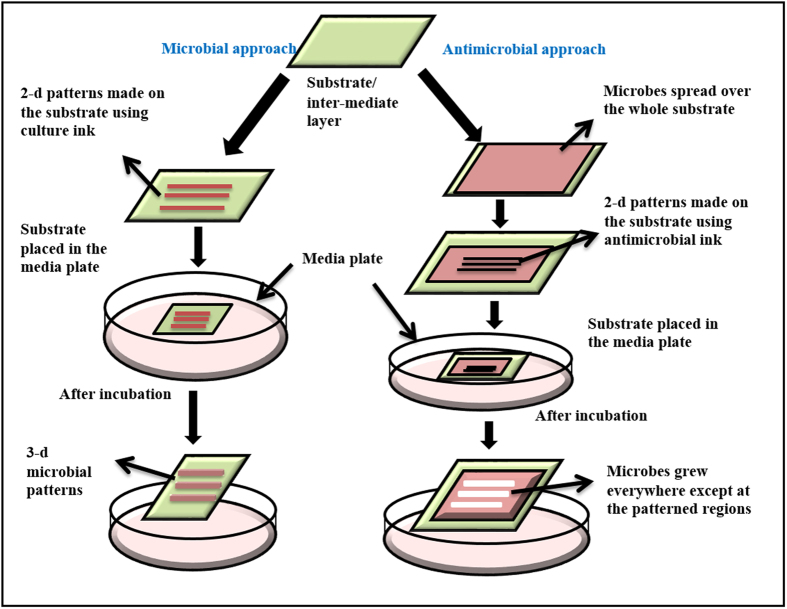
Schematic of the process developed for patterning of microbes. Microbial approach includes making 2d patterns of microbial culture on a permeable substrate, placing the substrate on the media plate and incubation; the 3d patterns of microbes are obtained on the substrate. Anti-microbial approach involves spreading microbial culture over the entire substrate and making patterns of antimicrobial agents where microbial growth is required to be restricted. Here also, the 3d patterns are generated by growth of microbes during incubation.

In the microbial approach, the microbes are directly inoculated in selected regions of the substrate. Localized growth in these regions leads to the formation of patterns (microbial approach, see Fig. 3). The extent of localization of growing patterns depends on the spreading of the inoculating solution on the membrane, which in turn, defines the pattern’s dimensions and resolution. In the case of RC membrane, the growth of microbial patterns is as expected. However, PVDF membrane requires functionalization. There can be two ways to functionalize. In one, the PVDF membrane is treated with surfactant first, making its surface completely hydrophilic (see Supplementary Fig. S1b online), and then dispensing the microbes in patterns (see Fig. 2c). Alternatively, since the surfactant is a growth enabler, a better option may be to include it with the microbial culture itself (“termed as culture ink”). When a mixed solution of microbial culture and Triton X-100 (10% by volume) is prepared (refer Methods for culture ink) and dispensed manually on PVDF membrane by a micropipette in form of letters “BSBE”, the microbes grow only on the dispensed regions (see Fig. 2d). Thus, whether the surfactant is spread on the membrane and then the inoculum is introduced, or surfactant is incorporated in the inoculum itself, both lead to microbial growth. This approach in Fig. 3 is the microbial approach.

In the second method called the antimicrobial method, microbes in form of culture ink (containing microbes and surfactant in solution) are inoculated throughout the membrane and then antimicrobial agents are dispensed in regions where growth is not needed. Growth of microbes occurring in all other regions forms the required pattern (antimicrobial approach, see Fig. 3). In this case, spreading of the anti-microbial agents on the membrane substrate would define the quality of patterns.

Among the different antimicrobial agents, organic solvents are selected because of their ability to inhibit microbial growth by causing permeabilization of cell membrane, which in turn leads to loss of cell integrity^18^. Different solvents such as ethanol, methanol, chloroform and iso-amyl alcohol were tested and iso-amyl alcohol was found to be the most suitable solvent (see Supplementary Fig. S2a online). Accordingly, when iso-amyl alcohol was dispensed on culture-ink coated PVDF membrane in the same pattern, that is, as letters ‘BSBE’, the growth of microbes upon incubation was inhibited (see Fig. 2e) in the dispensed region, leading to a negative replica of Fig. 2d.

Ultimately the membrane and pattern thus grown would have to be dried. But, upon drying, RC membrane crumples, leading to the loss of structural integrity of microbial patterns over its surface. On the other hand, PVDF retains its structural integrity, but the height of grown colonies of *E.Coli* and *E.Faecalis* was lost completely, possibly due to large inter and intra cellular water content in their colonies^19^. In the case of *Saccharomyces cerevisiae*, which is larger than bacteria (5–10 μm) and forms larger colonies, not only the growth of colonies occurred to a significant height (see Fig. 2c), most of it was retained when dried with a media layer underneath (see Fig. 2f). In summary, the initial selection of microbes was bacteria (*E.coli* and *E.faecalis –* both unicellular prokaryotes) because of their fastest doubling times and being routinely used in a biological laboratory for fundamental research. But, later, these bacteria were substituted with, yeast (unicellular fungus which is eukaryotic) to avail the desired significant third dimension even after drying. Hence, considering the uniformity of pattern formation and their processability, yeast patterns on PVDF was chosen as the best candidate for optimizing and developing patterns for use in engineering applications.

Thus two approaches for generating 3D patterns that produce growth in complimentary regions (compare Fig. 2d,e) have been demonstrated. However, we have developed the process by manual dispensing, which lacks the lateral dimensional control required for engineering applications. Therefore, manual dispensing is to be replaced by printing methods for better control. In the following, we demonstrate two distinct printing methods: ink-jet printing, with which we fabricate microlenses for light extraction and screen printing which is employed for producing braille text.

## Applications: engineering opportunities

We demonstrate the two engineering applications. First, a microbial pattern as an alternative to photo-lithographically prepared silicon master for microlens fabrication and then the potential of replacing mechanical production of pattern in Braille with microbes.

### Alternative to lithographic process: micro-lenses for enhancing light extraction from OLEDs

Micro-lenses deployed with organic light emitting diodes (OLEDs) help in extracting the glass mode of light by widening the escape cone for total internally reflected light incident at the air–glass boundary^14^. The maximum extraction efficiency depends upon the parameters such as lens diameter, height, shape, as well as the packing density of these microlens arrays. Various techniques have been employed for the fabrication of these micro-lenses. Among them photolithography is commonly used for patterning silicon masters that are employed for PDMS casting.

We demonstrate that the microbial patterns generated through the proposed process can be used for fabricating masters to prepare micro-lenses. Further the manner in which yeast grows presents several opportunities to tailor the shape and dimension of the micro-lenses. Inkjet printer is utilized for precise dispensing of the inhibitor or culture ink, corresponding to the two approaches in Fig. 3.

**Figure 4:**
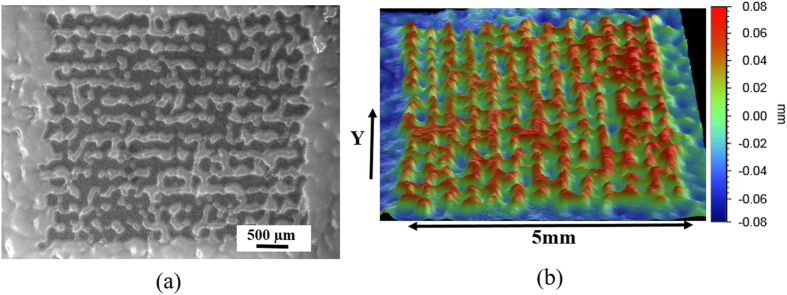
Micro-lenses fabricated using antimicrobial approach. (**a**) Optical image of microbial patterns obtained on PVDF membrane (**b**) 3d profilometer scan of PDMS microlenses prepared from microbial pattern using replica molding.

Using the antimicrobial approach, iso-amyl alcohol, the inhibiting agent, is printed in a hexagonal pattern of squares on PVDF membrane coated with culture ink (see Supplementary Fig. S2b online). After 24 hours of incubation, microbial growth is observed everywhere on the membrane except printed regions (see Fig. 4a). Although, the pattern is not reproduced rigorously due to uncertain spreading of iso-amyl alcohol on the random structure of pores on PVDF surface, yet it lends itself to preparing PDMS micro-lenses using replica molding (see Fig. 4b) (see “Fabrication of PDMS micro-lenses from microbial patterns” in Methods).

**Figure 5:**
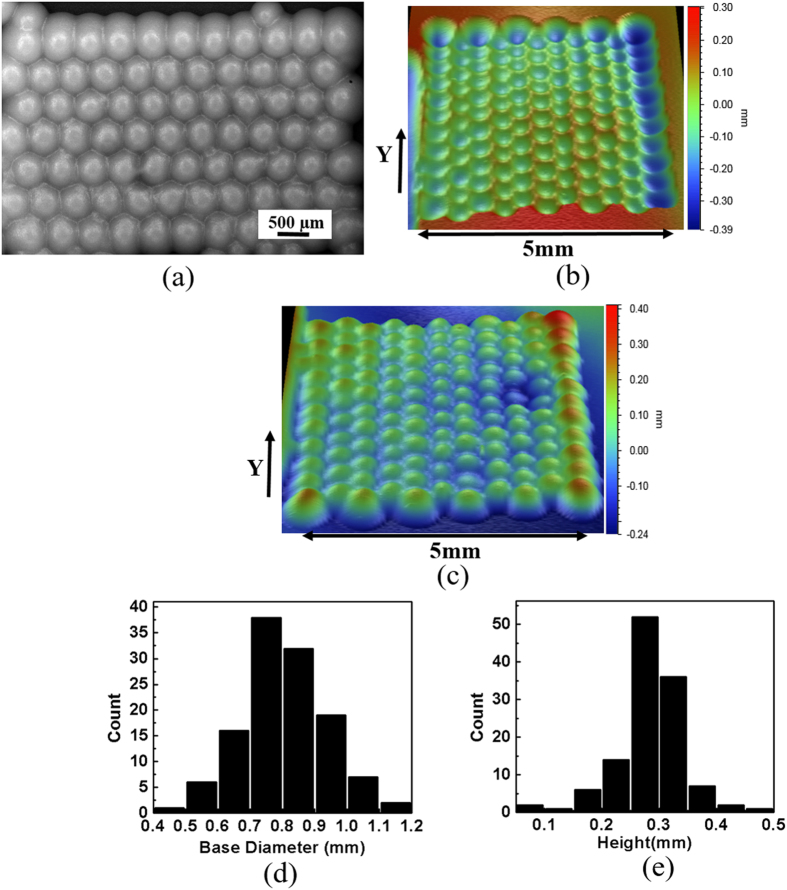
Micro-lenses fabricated using microbial approach. (**a**) Optical image of microbial patterns obtained on PVDF membrane after 36 hours of growth. (**b**) 3d profilometer scan of negative PDMS replica of microlenses prepared from microbial patterns; this serves as the master. (**c**) Positive PDMS replica of microlenses prepared from negative one after a surface treatment. This approach has resulted into more closely packed arrays of microlenses. The lenses are analyzed in form of conic sections. (**d**,**e**) are distributions of base diameter and height of the cones, respectively.

On the other hand, in the microbial approach, hexagonal patterns of squares of culture ink printed on the PVDF membrane (see Supplementary Fig. S3 online) merge into each other after growth; the microbes dispensed in form of 50 μm squares grow into circular colonies of size up to 500 μm and acquire a circular cross-section (see Fig. 5a). These microbial patterns allow for PDMS casting, which results into their negative replica (see Fig. 5b). Further, the negative replica thus prepared can be employed for second PDMS molding to get the microlens arrays (see Methods and Fig. 5c).

Based on line profiles of each of the columns of the lens array (see Supplementary Fig. S4a online), analysis suggests that these microlenses are in the form of interpenetrating paraboloids having nearly the same center to center distance (500 μm) as set while printing.

Accordingly, the base diameter, defined as notional diameter to which an individual colony would have grown, and the height of the conic form of lenses are distributed according to Fig. 5d,e. The distributions are characterized by average base diameter 810 μm (standard deviation of 127 μm) and average cone height 284 μm (standard deviation 57 μm).

**Figure 6:**
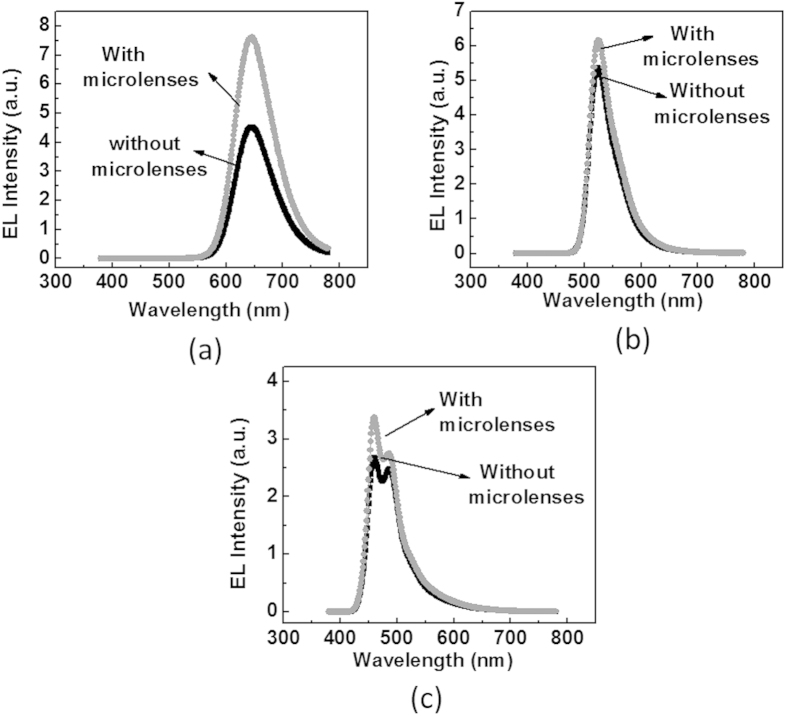
Electro-Luminance spectra for OLEDs. Electro-luminance spectra obtained for (**a**) red (**b**) green and (**c**) blue OLEDs without as well as with micro-lenses. No shift in the peak suggest that micro-lenses do-not affect the spectra for all three devices.

To demonstrate the applicability of microlens arrays thus prepared, they were attached to the glass surface of red, green and blue OLEDs separately. Compared to OLEDs without lenses, the luminance enhancement (see table 1) with lenses formed by microbial approach was better than that by antimicrobial approach for all red, green and blue OLED devices. The maximum enhancement of 1.24X, was even better than that achieved from lenses molded from silicon master in our laboratory with same PDMS material. It was also seen that these micro-lenses do not affect the spectra of the corresponding OLEDs (see Fig. 6).

In the lenses prepared using microbial approach, the microbes dispensed at a center to center separation of 500 μm begin to grow laterally more rapidly than vertically, impinging into each other. That results into notional base diameters exceeding the center to center distance (see Supplementary Fig. S4b online). On top of the base formed by impinged colonies of the microbes are small conic sections (fitted to parabolas in line scans) with a height of the order of 300 μm (local protrusions are of the order of 100 μm). The shape of these cones is somewhat flat with larger diameter, affording an opportunity to further optimize the height to diameter ratio. Nonetheless, due to the nature of the microbial growth, these lenses are closed packed. In the literature related to light out-coupling from GaN:LEDs, close packed conical structures have been reported to be optimal antireflective structures because of the refractive index gradient formed at the air-substrate interface^20^. Similarly, among various aspherical micro-lenses prepared by multistep lithography, the best curve profile for extraction of light was a parabola^21^. Here we have shown that not only the lenses formed by microbial growth have parabolic curve profile and are well packed, but they are also random in both diameter and height, which affords even better performance^22^.

### Alternative to mechanical 3-d patterns: braille printing

To obtain a tactile feel, braille patterns must have a minimum embossed dimension of 500 μm. Such patterns are generally produced by mechanical pressing of embossing pins on a specialized, thus expensive, paper that is thick so that it can retain deformation. The objective here is to replace such paper with ordinary paper. Since the microbial colonies typically have lateral dimensions in the range of millimeters, an application for microbial patterns can be envisaged in braille printing. To this end, screen printing has been employed to print the 2D yeast patterns on a sterilized 75 GSM office paper (see Supplementary Fig. S5 online and Methods), instead of PVDF membrane used earlier.

The ordinary copying paper that was used predominantly contains cellulose and hemi-cellulose fibers. It therefore allows sufficient permeation of media components through it. Special care was taken to prevent crumpling upon drying (see Methods).

**Figure 7:**
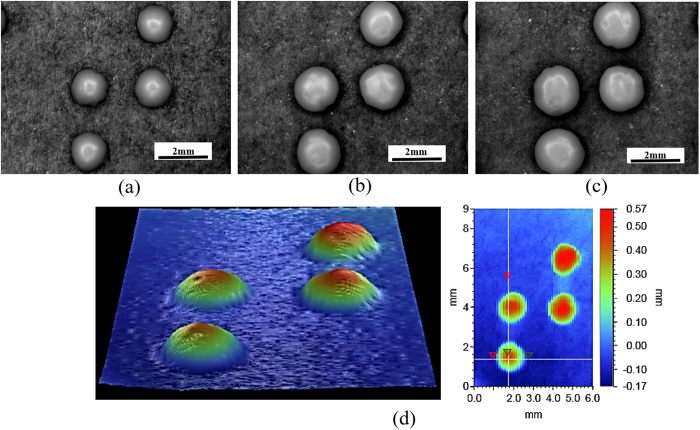
Characterization of Braille dots prepared using microbes. Optical image of yeast colonies in the pattern of letter “T” in Braille text on A4 paper grown to diameters (**a**) 1.4 mm (**b**) 1.6 mm (**c**) 2.0 mm. The corresponding diameter on the screen in the same order was 0.23, 0.43 and 0.63 mm. (**d**) 3d profilometer scan of Braille dot (from diameter = 0.43 mm). Height obtained of raised dot was 0.5 mm, closely matching a Braille standard. (The braille texts thus prepared were also checked with visually impaired people for readability).

The screen employed for printing contains dot patterns of three different diameters 0.23 mm, 0.43 mm and 0.63 mm (see Supplementary Fig. S6 online). Accordingly, after incubation the grown yeast dot patterns resulted into the diameters of 1.4 mm, 1.6 mm and 2 mm (see Fig. 7a–c), respectively. These patterns are then dried and preserved with a thin layer of lacquer introduced through spin coating. Amongst the three dot diameters, the one with the final grown diameter of 1.6 mm and height 0.5 mm is closest to the standard dot requirements for printed braille books. Figure 7d confirms the lateral and vertical dimensions of the dots obtained after drying. Also note that, the dots have grown in diameter by 1 to 1.4 mm. Though the substrate and ink are different, the increase in diameter upon incubation is similar to the notional base diameter in the case of microlenses. Finally, we are able to demonstrate that indeed, unlike conventional mechanical printing of braille dots, special paper to retain the prints is not needed.

## Conclusions and Discussion

Inspired by a tree, we have successfully employed commonly available microbes for the fabrication of three dimensional patterns of wide dimensional range (from few hundreds of microns to millimeter). Further, the use of intermediate porous substrate enables these microbial patterns for many engineering applications. Such patterns are obtained either by patterning the grown region (microbial approach) or by limiting the growth of microbes in certain regions (antimicrobial approach). Equivalent to the latter can also obtained by blocking the pores of the membrane where growth is not required or using an impervious substrate with 2D patterns cut into it. (We already demonstrated those equivalent processes by blocking the membrane pores using paint printing or by utilizing plastic sheet with laser cut 2D features. Either of these are then directly kept on the gel media so that growth is possible only in the cut/unblocked regions^23^). Based on the process, variety of engineering applications are possible, such as using the microbial pattern directly as stamps in contact printing, or preparing a stamp, such as PDMS stamp, for subsequent printing. We have succeeded to some extent in printing with the stamps thus made. In order to demonstrate the variety of possible applications, we have selected an example where patterns are made in silicon by etching for microlenses and another where patterns are made mechanically, as in Braille printing.

The importance of the present work lies in that, in general,

(1) we use common yeast, which is non-pathogenic, eco-friendly and inexpensive,

(2) the starting two dimensional pattern can be produced by low cost capital equipment,

(3) in the method presented, only two dimensional pattern is made and, unlike the conventional methods, the third dimension (height) is achieved by utilizing the natural ability of microbes to grow when provided with sufficient nutrients,

(4) currently the lateral and vertical dimension achievable is in the range of hundreds of microns,

(5) smaller lateral resolution is possible by conventional methods, but producing the third dimension requires an additional process step, and also use of harsh chemicals such as in etching. But here, since that step is avoided, we also avoid the use of harsh chemicals and

(6) the processing steps involved are extremely simple and easy to perform.

In addition, the conventional method will be limited to small sizes. Specific advantage of the method presented here, however, is that it can potentially be applied in large sizes, for example in lenses for OLED and Braille printing. Also, Braille printing employs specialized and expensive printers, along with a special thick paper that is expensive, and mechanical impressions in paper tend to diminish in height during use. In contrast, the present work uses ordinary office paper and patterns that are likely to remain stable in time.

However, there are still many challenges to be resolved in order to realize the full potential of the invention. The major ones being the dimensional control and the time required for the completion of the process. For this, first, either the microbes or the growth conditions are to be changed, so that time scale of the growth is significantly reduced, from hours to minutes. Further, the 2D features demonstrated are in 100 microns range. In order to make the process applicable to electronic devices by micro-contact printing, the dimensions would have to be brought down to smaller sizes, potentially by replacing polymeric membranes with other smoother surfaces such as porous polished ceramics. In other words, the microbes would have to be smaller and their colonies made denser. However, it is unlikely that dimensions can be sub-micrometers. In addition, there is an issue of lateral variation in dimensions. This is, however, also related to obtaining small dimensions; that is, this variation would also be small when small features can be printed. Nonetheless, even presently, the issue of dimensional control is not significant when dealing with the features in the millimeter range as in braille patterns. Additional challenge may be due to roughness of the top surface of the grown structure. In OLEDs, this roughness could be considered an advantage. However, for applications requiring smoother grown surfaces, further development is required, potentially by similar ideas as those required for creating smaller dimensions. Further, in context of Braille printing, the method presented would be good for custom small volume printing. However, then instead of screen printing of microbes that has been used for demonstration, a mechanized dispenser would serve the purpose.

## Materials and Methods

### Microbes

*Saccharomyces cerevisiae, Escherichia coli* (DH5-α) *and Enterococcus faecalis* (ATCC 19433).

### Substrates

Regenerated cellulose membrane (pore size 13 kDa) purchased from Sigma Aldrich, Polyvinylidene fluoride (pore size 0.45 μm) membrane purchased from Millipore and 75 GSM office paper.

### Other materials

Yeast Peptone Dextrose (YPD), Lurani-Bertoni (LB) and agar all from Hi-media, Triton X-100 (SD-Fine Chemicals), iso-amyl alcohol (SD-Fine Chemicals), PDMS (Dow-corning Sylguard).

## Methods

### Preparation of media plate

An aqueous solution of powdered 5 gms YPD (for yeast) or LB media (for *E.coli and E.faecalis*) with 2 gms of gelating agent (agar) in 100 ml distilled water was sterilized and poured in petri-plates while avoiding inclusion of air-bubbles. After some time it forms a layer of gel, which served as suitable surface for microbial growth.

### Preparation of inoculant solution

A small quantity of inoculating microbes was introduced in the sterilized aqueous solution of YPD or LB media in a test tube using sterilized loop/tip in a sterilized environment and was incubated at 37 °C for a duration depending upon the microbes used.

### Use of RC membrane as a substrate for microbial growth

RC membrane was autoclaved and placed in the media plate. Microbes were streaked on it using sterilized loop/tip. Plate was kept in the incubator for overnight (for *E.coli and E.faecalis*) or 24 hrs (for yeast).

### Use of PVDF membrane as substrate for microbial growth

PVDF membrane was dipped in the sterilized surfactant solution for 3–4 hours. Membrane was blotted against tissue paper to remove extra surfactant on its surface and then kept on media plate for 15–20 minutes for drying. After drying, microbes were streaked using sterilized loop or printed on the membrane with a media layer underneath and then plate was kept in the incubator for overnight (for *E.coli and E.faecalis*) or 24 hrs (for yeast).

### Use of organic solvents as antimicrobial agent

For this, a culture (inoculant solution) was grown to an optical density (OD_600_) of 1 and then spread over the surfactant treated PVDF membrane. The antimicrobial agent was dispensed over this membrane kept on a media layer and then the media plate was kept in the incubator for 24 hours.

### Inkjet Printing

For precise dispense of culture ink or antimicrobial agent, a Dimatix printer with piezo driven print-head was used. In microbial approach the culture ink was optimized in order to achieve surface tension and viscosity values compatible for Dimatix printer (σ = 25–35 mN/m and η = 1–8 cP). A suitable ink termed as “culture ink” was obtained by adding 10% (v/v) triton X-100 in the yeast culture of OD_600_ = 1 (optimization parameters are shown in the Supplementary Fig. S3 online). This ink was printed on the PVDF membrane placed on a media layer and kept in the incubator for 36 hours. For antimicrobial approach, the selected iso-amyl alcohol already had surface tension and viscosity values compatible with Dimatix printer. This ink was also printed on PVDF membrane.

### Fabrication of PDMS micro-lenses from microbial patterns

PDMS solution was prepared by mixing base and curing agent in the ratio of 10:1 (w/w) and desiccating the solution for about an hour to remove air-bubbles generated during mixing. The solution was then poured directly over wet microbial patterns and cured at 60 °C for 2 hours. After cooling to room temperature, PDMS was peeled off from PVDF, washed with DI-water to remove any attached microbes and dried gently using nitrogen gun. In antimicrobial approach, such PDMS sheets directly served as the microlenses. However, in microbial approach, this PDMS sheet was used as a master. Before another PDMS replica molding, this master was first ozonized for easy removal of subsequent PDMS sheets.

### Screen printing for preparing microbes based braille text

The screen (mesh count 300/inch) consisted of dots (corresponding to letter “T” written in braille) of diameters 0.23 mm, 0.43 mm and 0.63 mm. The ink preparation involved centrifugation of microbial culture at 4500 rcf for 15 minutes, washing with Tris buffer (pH = 7.6) next and then again centrifugation at 4500 rcf for 15 minutes. After decanting the supernatant, surfactant (20% w/w) was added to this biomass; this served as the screen printing ink. After screen printing on the paper, the paper was placed on the media plate before incubation for 32 hours.

### Rigidifying microbial patterns

After incubation of the screen printed yeast patterns, these grown microbes were rigidified by dehydrating the moisture content in the cells at 80 degrees for 3 hours. However, during drying the media layer was required underneath the paper in order to avoid cracks in the dried microbial patterns. These dried patterns were further preserved by coating a thin layer of lacquer over these patterns. After this preservation step, underneath media layer was wiped off.

### Fabrication of light Emitting Diodes (LEDs)

The red OLED stack on glass was ITO (150 nm)/DS205(100 nm)/NPB(20 nm)/Alq3 (19.8 nm)+rubrene (13.2 nm)+TC1712 (1.7%)/Alq3 (30 nm)/LiF(0.8 nm)/Al(150 nm)/NPB (50 nm). The green stack was ITO (150 nm)/DS205(100 nm)/NPB(20 nm)/DS H522 (30 nm)+DS 501 (3%)/Alq3 (30 nm)/LiF(0.8 nm)/Al(150 nm)/NPB (50 nm). The blue stack was ITO (150 nm)/DS205(100 nm)/NPB(20 nm)/DSH 43(30  nm)+DS 405 (10%)/Alq3 (30 nm)/LiF(0.8 nm)/Al(150 nm)/NPB (50 nm).

For all red, green and blue OLEDs on indium tin oxide (ITO) glass, a hole injection layer (DS205) was followed by NPB (N, N′–diphenyl–N, N′–bis (1, 1′-biphenyl)–4, 4′–diamine). The emissive layers were deposited next. The red emissive layer consisted of co-evaporated Alq3 (Tris-(8-hydroxyquinoline) aluminum) host, rubrene co-host and TC1712 dopant, green emissive layer was host DS H522 coevaporated with dopant DS 501 and blue emissive layer was host DS H43 and DS 405 dopant. Following the emissive layers, stack was identical for all red, green and blue OLEDs. It consisted of Alq3, LiF and Al which was capped by NPB. DS205 was hole injection layer (HIL), DS H522 was green host, DS 501 was green dopant, DS H43 was blue host and DS 405 was blue dopant all from Doosan Electromaterials, TC1712 was red dopant supplied by TTC (Tetrahedron Technology Corporation). NPB and Alq3 were supplied by Sensient Technologies USA, Rubrene was provided by e-ray optoelectronic technologies Taiwan.

## Additional Information

**How to cite this article**: Mehta, S. *et al.* Fabrication of three dimensional patterns of wide dimensional range using microbes and their applications. *Sci. Rep.* **5**, 15416; doi: 10.1038/srep15416 (2015).

## Supplementary Material

Supplementary Information

## Figures and Tables

**Figure 1:**
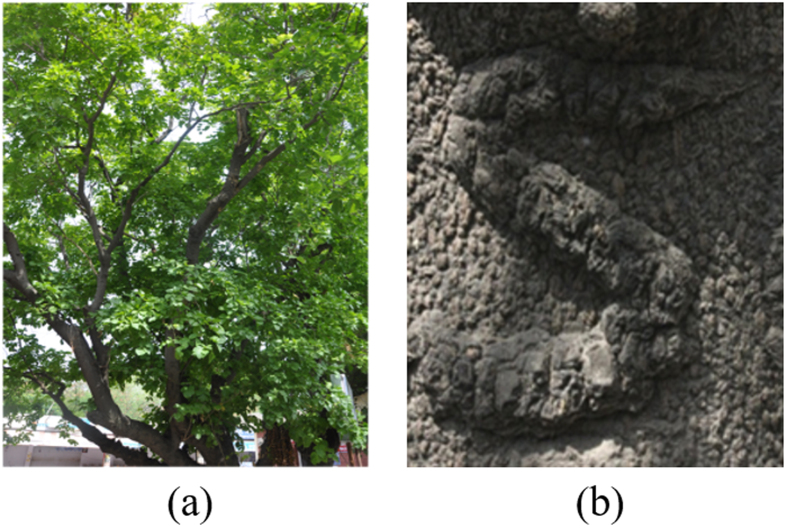
The inspiring tree. (**a**) Photographic image of *Pongamia Pinnat*a. (**b**) letter S scratched on the trunk of this tree appears as a protrusion after recovery; in most other trees, this forms an indentation instead.

**Table 1:** Results of Luminance Measurements on LED devices.

LED	% Enhancement	
	With Microbialapproach	With antimicrobialapproach
Red	24	12
Green	18	11
Blue	19	10
