# Supplementary material for: Fabrication of three dimensional patterns of wide dimensional range using microbes and their applications
Source: Sci Rep. 2015 Oct 21;5:15416. doi: 10.1038/srep15416 (PMC4613880; doi:10.1038/srep15416)

Supplementary Information

Fabrication of three dimensional patterns of wide dimensional range using microbes and their applications

Sunita Mehta[1], Saravanan Murugeson[2], Balaji Prakash[2†], Deepak[1*]

[1] Department of Materials Science & Engineering and Samtel Center for Display Technologies, Indian Institute of Technology Kanpur, Kanpur-208016, India.

[2] Department of Biological Sciences & Bioengineering, Indian Institute of Technology Kanpur, Kanpur-208016, India.

[†] Present Address- Department of Molecular Nutrition, CSIR-Central Food Technological Research Institute, Mysore-570020, India.

Supplementary Materials:
Supplementary Figures S1–S6

[*] Deepak
Professor
Department of Materials Science & Engineering
& Samtel Centre for Display Technologies
Indian Institute of Technology Kanpur
Kanpur, UP-208016, India
E-mail: saboo@iitk.ac.in

Supplementary Figure S1

Effect of surfactant treatment on PVDF membrane. Contact angle measurements were performed using sessile drop method to check the wettability of PVDF membrane with and without treatment of Triton X-100. Water was used as a test liquid. Pristine PVDF membrane shows a static contact angle of 137±2^0 (Fig. a). For PVDF treated with 0.1%(v/v) Triton X-100, dynamic contact angle measurements were performed and it was found that wettability was increased to an extent that water contact angle decreases from 23^0 ±1^0 to 4^0 ±1^0 and then finally drop was completely absorbed in the membrane (Fig. b); the contact angle value in itself may not be accurate, but it indicates change in the nature of the surface.

a

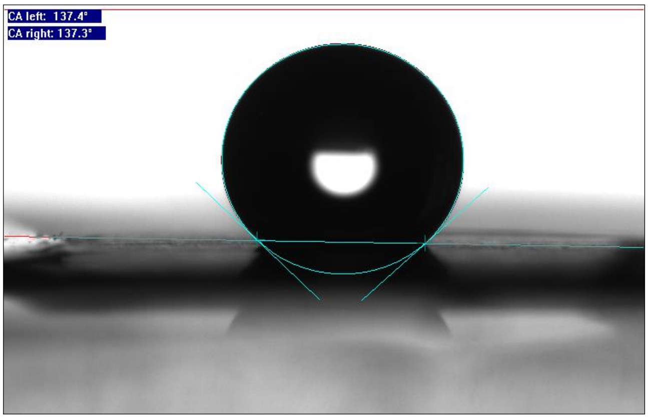

b

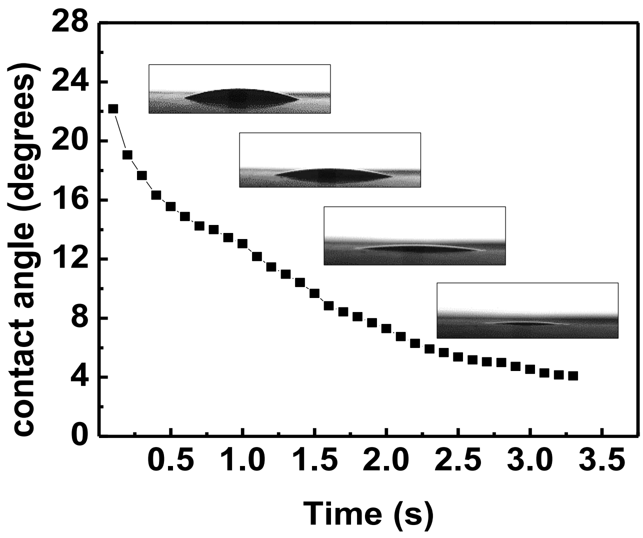

Supplementary Figure S2

Selection of antimicrobial agent. In order to test inhibiting activity of organic solvents (ethanol, methanol, chloroform and iso-amyl alcohol) ,these solvents were introduced in the liquid media being used for inoculation and microbial growth was examined in terms of optical density measured at 600 nm(OD_{600}) after 24 hours incubation.

OD_{600} values in the presence of these solvents being significantly lower than that for culture approved them as antimicrobial agents (Fig. S2a). Hence, all four show antimicrobial activity, but among these, iso-amyl alcohol was selected because of its stability (high boiling point 131^0C) and suitable surface tension and viscosity values; the surface tension of all four is in acceptable range of 22-26 mN/m but viscosity of iso-amyl alcohol is more compatible with drop on demand inkjet printer used for printing these chemicals. Fig. b shows the jetting of iso-amyl alcohol from cartridge nozzles at V=12V and Frequency=10 KHz. Squares (100 µm) of iso-amyl alcohol with center to center separation of 300 µm were printed in a hexagonal pattern on PVDF membrane.

a

Table: Optical Density (OD_{600}) values measured after 24 hours of growth

	Control (media)	culture	ethanol	methanol	chloroform	Iso-amyl alcohol
OD_{600}	0	1.022	0.021	0.126	0.117	0.097

b

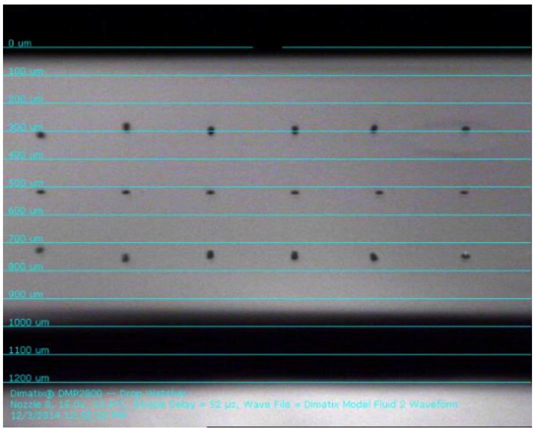

Supplementary Figure S3

Ink preparation for microbial approach. (a) Surface tension and viscosity values for varying concentrations of Triton X-100 added to yeast culture of OD_{600}=1 (b) jetting of culture ink (yeast culture +10%v/vTriton X-100) from catridge nozzles at V=18V and Frequency=10 KHz. The squares (50 µm) of culture ink with center to center separation of 500 µm were printed in a hexagonal pattern on PVDF membrane.

a

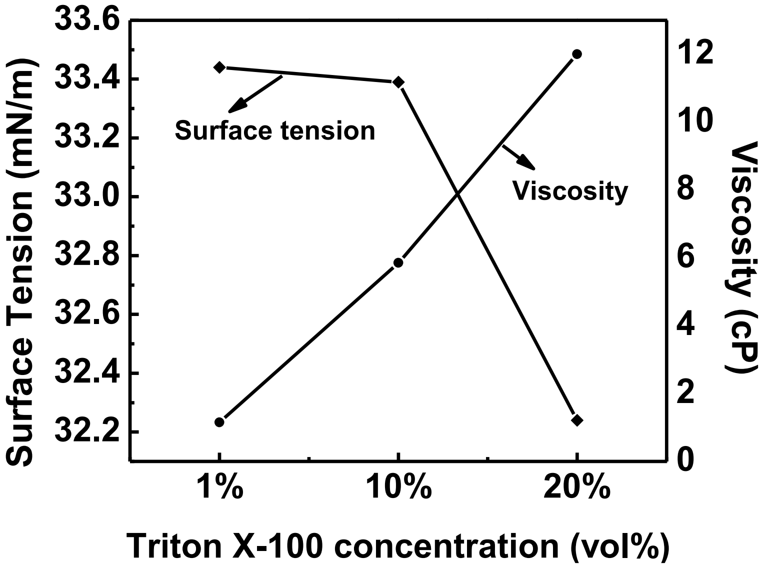

b

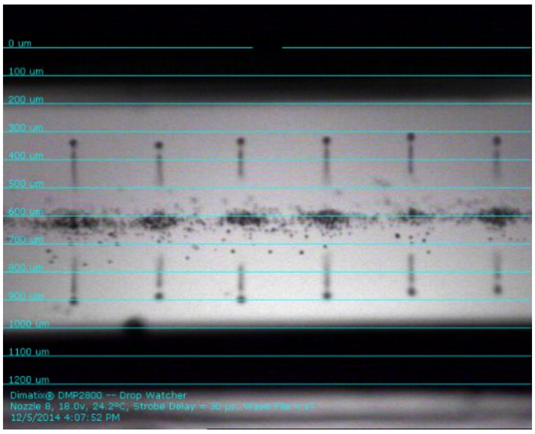

Supplementary Figure S4

Analysis of microlenses prepared using microbial approach. The lens array shown in Fig. 5(c) of the main paper consists of 11 linear columns of lenses. A profilometer line scan through the center of a lens is taken for each column. A typical line scan of lens height (Z) is shown in Fig. (a). In (b), the line scan is leveled at the two ends and the lens clearly appears to be conic in shape. A parabola fits well to each of these lenses, as shown in Fig. (b). Hence, lenses seem to form by interpenetrating conic sections. From the base (zero Z level), the microbes corresponding to a lens have grown to a height noted by the cone tip. This is called the height of the lens. The base diameter, that is, at Z=0, also the level at which the microbes were originally dispensed, is extracted from fitting of the parabolas. These base diameters are as large as approximately 1 mm. The center to center distance of each parabola is approximately 500 µm, the same as the separation at which microbes were dispensed. This suggests isolated squares of 50 µm would have grown to diameters of the order of 1 mm, which then can be regarded as the notional base diameter of the lenses.

a **b**

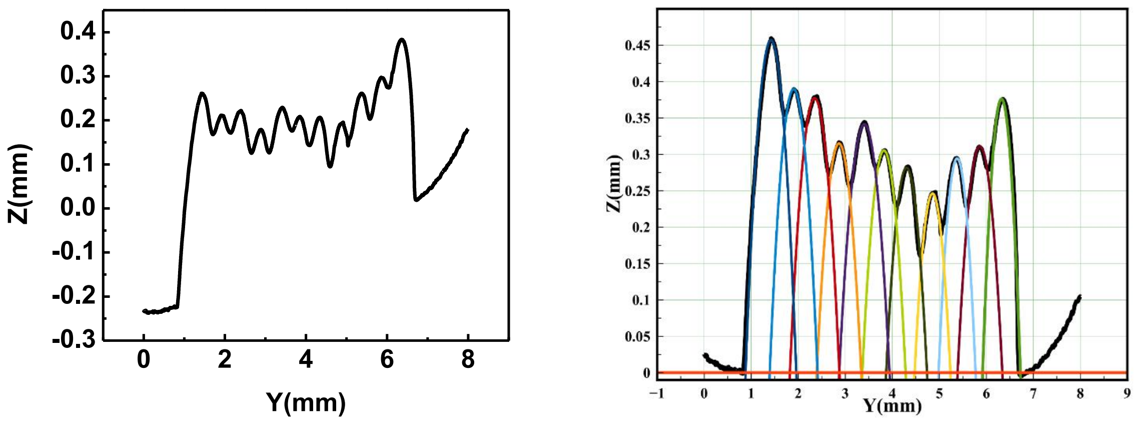

Supplementary Figure S5

Ink for Screen printing. Fig. (a) shows shear thinning behavior of the ink prepared for screen printing. The decrease in viscosity of ink during squeeze stroke (shear rate › 1000/s), allows the ink to flow through the screen easily and as soon as it reaches to the substrate (at low shear rate) it regains its high viscosity value.

a

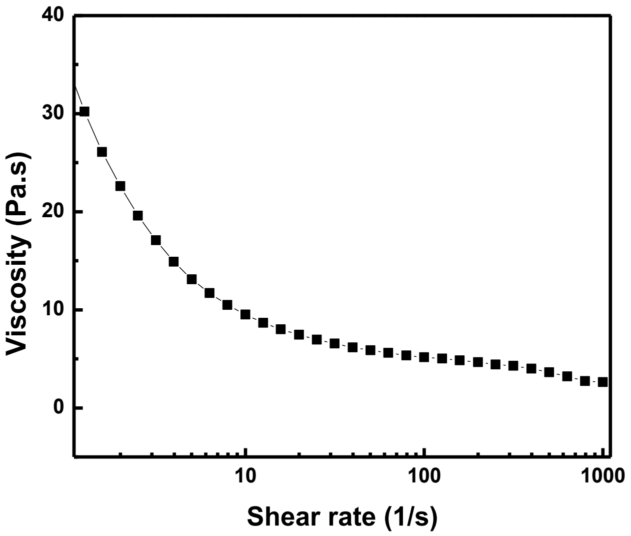

Supplementary Figure S6

(a) Dot patterns on screen with diameter =0.23mm, 0.43mm and 0.63 mm respectively. (b) Variation of as grown yeast diameter as a function of time for three different dot diameters on the screen. After 32 hours, yeast has grown upto diameter of 1.4, 1.6 and 2.0 mm with dot diameters of 0.23 mm, 0.43 mm and 0.63 mm respectively.

a

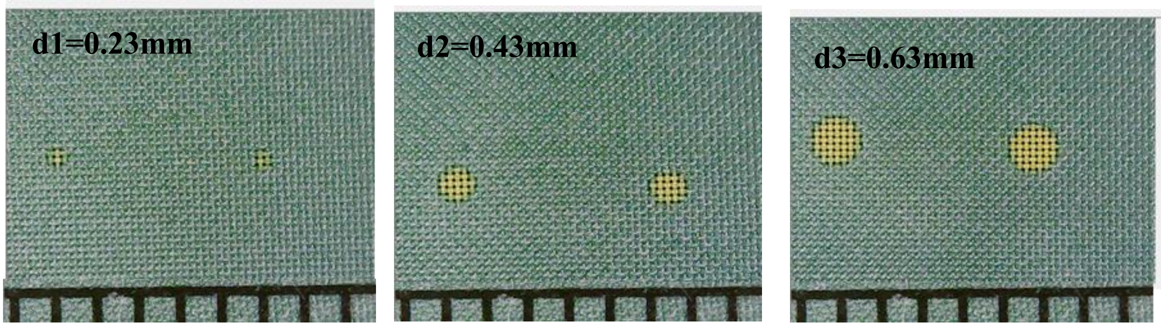

Scale: 1 div=500 μm

b

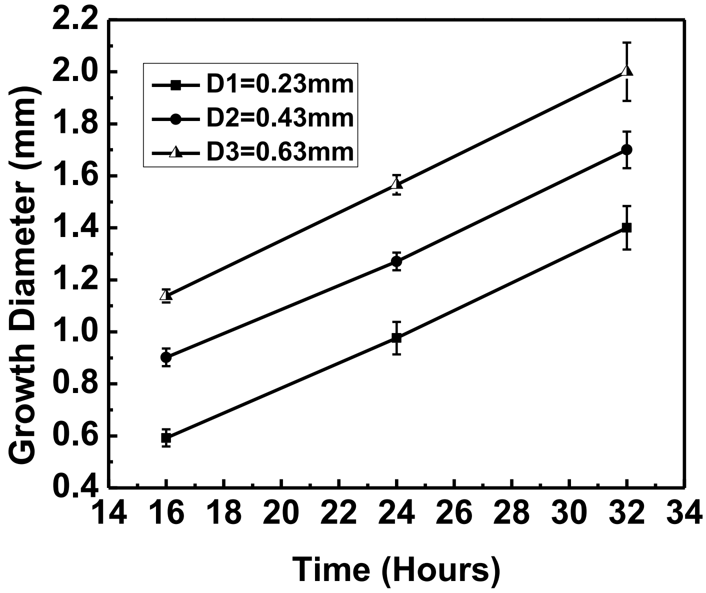

Supplement: Supplementary Information [file srep15416-s1.pdf]
